# Erratum: Meyer, J., et al. Changes in Physical Activity and Sedentary Behavior in Response to COVID-19 and Their Associations with Mental Health in 3052 US Adults. *Int. J. Environ. Res. Public Health* 2020, *17*(18), 6469

**DOI:** 10.3390/ijerph17196949

**Published:** 2020-09-23

**Authors:** Jacob Meyer, Cillian McDowell, Jeni Lansing, Cassandra Brower, Lee Smith, Mark Tully, Matthew Herring

**Affiliations:** 1 Department of Kinesiology, Iowa State University, Ames, IA 50011, USA; jenil@iastate.edu (J.L.); cbrower@iastate.edu (C.B.); 2 The Irish Longitudinal Study on Aging and School of Medicine, Trinity College Dublin, The University of Dublin, D02 R590 Dublin, Ireland; Cillian.McDowell@tcd.ie; 3 Cambridge Centre for Sport and Exercise Sciences, Anglia Ruskin University, Cambridge CB1 2LZ, UK; Lee.Smith@anglia.ac.uk; 4 Institute of Mental Health Sciences, Ulster University, Coleraine, Northern Ireland BT37 0QB, UK; m.tully@ulster.ac.uk; 5 Physical Activity for Health Research Cluster, Health Research Institute, Department of Physical Education and Sport Sciences, University of Limerick, V94 T9PX Limerick, Ireland; Matthew.Herring@ul.ie

Due to an error during production, Dr. Meyer and Dr. McDowell were not both listed as joint first authors in the article in the International Journal of Environmental Research and Public Health [1]. The corrected author list is below.

Jacob Meyer ^1,^*^,†^, Cillian McDowell ^2,†^, Jeni Lansing ^1^, Cassandra Brower ^1^, Lee Smith ^3^, Mark Tully ^4^ and Matthew Herring ^5^

^1^ Department of Kinesiology, Iowa State University, Ames, IA 50011, USA; jenil@iastate.edu (J.L.); cbrower@iastate.edu (C.B.)
^2^ The Irish Longitudinal Study on Aging and School of Medicine, Trinity College Dublin, The University of Dublin, D02 R590 Dublin, Ireland; Cillian.McDowell@tcd.ie
^3^ Cambridge Centre for Sport and Exercise Sciences, Anglia Ruskin University, Cambridge CB1 2LZ, UK; Lee.Smith@anglia.ac.uk
^4^ Institute of Mental Health Sciences, Ulster University, Coleraine, Northern Ireland BT37 0QB, UK; m.tully@ulster.ac.uk
^5^ Physical Activity for Health Research Cluster, Health Research Institute, Department of Physical Education and Sport Sciences, University of Limerick, V94 T9PX Limerick, Ireland; Matthew.Herring@ul.ie
^*^ Correspondence: jdmeyer3@iastate.edu
^†^ These authors contributed equally to this work.

The authors would like to apologize for any inconvenience to the readers caused by this error. The article will be updated and the original will remain on the webpage.
