# Supplementary material for: Changes in Physical Activity and Sedentary Behavior in Response to COVID-19 and Their Associations with Mental Health in 3052 US Adults
Source: Int J Environ Res Public Health. 2020 Sep 5;17(18):6469. doi: 10.3390/ijerph17186469 (PMC7559240; doi:10.3390/ijerph17186469)
Supplement: Supplementary file 1 [file ijerph-17-06469-s001.pdf]

Table S1. Adjusted associations between COVID-19 related public health restrictions and mental health

		Depression		Anxiety		Loneliness		Stress		Social Network		Positive Mental health	
	n	b (SE)	*p*-value	b (SE)	*p*-value	b (SE)	*p*-value	b (SE)	*p*-value	b (SE)	*p*-value	b (SE)	*p*-value
**Public Health Restrictions**													
Social distancing	550	REF		REF		REF		REF		REF		REF	
Stay at home	1462	0.320 (0.297)	0.282	0.491 (0.285)	0.085	-0.019 (0.067)	0.778	0.169 (0.111)	0.127	0.220 (0.105)	0.035	-0.058 (0.162)	0.718
Self-isolation	1040	1.427 (0.424)	<0.001	1.640 (0.440)	<0.001	0.082 (0.090)	0.364	0.250 (0.146)	0.086	0.084 (0.142)	0.557	-0.171 (0.226)	0.451

Adjusted for age, sex, race, smoking, physical activity, sitting time, screen time, relationship status, employment, and chronic illnesses. Education was excluded due to multicollinearity. b = adjusted unstandardized beta; SE = standard error.
Statistical significance set at $p < 0.00833$

Table S2. Adjusted associations between changes in behavior from before to after COVID-19 related public health restrictions including covariates with mental health outcomes

		Depression		Anxiety		Loneliness		Stress		Social Network		Positive Mental Health	
	n	b (SE)	*p*-value	b (SE)	*p*-value	b (SE)	*p*-value	b (SE)	*p*-value	b (SE)	*p*-value	b (SE)	*p*-value
**Physical Activity**													
Maintained high	798	REF		REF		REF		REF		REF		REF	
Increased	152	-0.505 (0.645)	0.434	0.066 (0.677)	0.923	0.001 (0.136)	0.996	-0.133 (0.244)	0.585	0.305 (0.222)	0.169	0.189 (0.345)	0.585
Decreased	563	1.960 (0.417)	<0.001	0.596 (0.411)	0.148	0.340 (0.096)	<0.001	0.522 (0.155)	<0.001	-0.269 (0.149)	0.072	-1.010 (0.230)	<0.001
Maintained low	1539	0.629 (0.318)	0.048	0.248 (0.320)	0.439	0.078 (0.075)	0.302	0.377 (0.124)	0.002	-0.389 (0.119)	0.001	-0.629 (0.182)	<0.001
**Sitting Time**													
Maintained low	1041	REF		REF		REF		REF		REF		REF	
Decreased	85	0.587 (0.972)	0.546	1.497 (0.944)	0.113	-0.067 (0.197)	0.728	-0.015 (0.329)	0.964	0.074 (0.273)	0.786	-0.122 (0.505)	0.809
Increased	582	0.918 (0.441)	0.037	0.946 (0.445)	0.034	0.195 (0.097)	0.045	0.253 (0.154)	0.102	-0.163 (0.149)	0.275	-0.673 (0.234)	0.005

Maintained high	1344	-0.199 (0.348)	0.566	-0.064 (0.344)	0.852	0.046 (0.078)	0.554	-0.068 (0.131)	0.604	-0.040 (0.126)	0.750	-0.036 (0.192)	0.853
**Screen Time**													
Maintained low	1512	**REF**		**REF**		**REF**		**REF**		**REF**		**REF**	
Decreased	45	-0.623 (1.095)	0.569	0.345 (1.203)	0.774	-0.495 (0.198)	0.013	-0.261 (0.468)	0.577	0.762 (0.393)	0.052	0.412 (0.641)	0.520
Increased	562	1.924 (0.441)	<0.001	1.341 (0.454)	0.003	0.340 (0.095)	<0.001	0.590 (0.154)	<0.001	-0.069 (0.145)	0.632	-0.920 (0.239)	<0.001
Maintained high	933	0.375 (0.392)	0.339	0.474 (0.375)	0.206	0.146 (0.085)	0.087	0.126 (0.137)	0.361	-0.156 (0.133)	0.243	-0.451 (0.202)	0.026
**Public Health Restrictions**													
Social distancing	550	**REF**		**REF**		**REF**		**REF**		**REF**		**REF**	
Stay at home	1462	0.320 (0.297)	0.282	0.491 (0.285)	0.085	-0.019 (0.067)	0.778	0.169 (0.111)	0.127	0.220 (0.105)	0.035	-0.058 (0.162)	0.718
Self-isolation	1040	1.427 (0.424)	<0.001	1.640 (0.440)	<0.001	0.082 (0.090)	0.364	0.250 (0.146)	0.086	0.084 (0.142)	0.557	-0.171 (0.226)	0.451
**Age**													
18–24	508	**REF**		**REF**		**REF**		**REF**		**REF**		**REF**	
25–34	470	-2.940 (0.712)	<0.001	-2.067 (0.715)	0.004	-0.158 (0.134)	0.238	-0.487 (0.216)	0.024	-0.400 (0.200)	0.045	0.892 (0.333)	0.007
35–44	419	-3.506 (0.754)	<0.001	-3.184 (0.731)	<0.001	-0.289 (0.146)	0.048	-0.642 (0.241)	0.008	-0.524 (0.220)	0.017	1.230 (0.367)	0.001
45–54	376	-5.132 (0.762)	<0.001	-4.211 (0.727)	<0.001	-0.466 (0.148)	0.002	-1.204 (0.244)	<0.001	-0.448 (0.229)	0.051	2.032 (0.374)	<0.001
55–64	474	-7.497 (0.731)	<0.001	-5.997 (0.694)	<0.001	-0.518 (0.149)	0.001	-1.793 (0.240)	<0.001	-0.465 (0.225)	0.039	3.386 (0.365)	<0.001
65–74	522	-8.571 (0.790)	<0.001	-6.299 (0.757)	<0.001	-0.604 (0.165)	<0.001	-2.374 (0.278)	<0.001	-0.386 (0.259)	0.136	4.444 (0.428)	<0.001
75+	283	-8.701 (0.822)	<0.001	-6.093 (0.795)	<0.001	-0.885 (0.189)	<0.001	-2.159 (0.315)	<0.001	-0.688 (0.291)	0.018	4.966 (0.489)	<0.001
**Sex**													
Male	1151	**REF**		**REF**		**REF**		**REF**		**REF**		**REF**	
Female	1897	1.658 (0.284)	<0.001	3.323 (0.259)	<0.001	0.218 (0.065)	0.001	0.622 (0.106)	<0.001	0.627 (0.104)	<0.001	-0.947 (0.163)	<0.001
Transgender	4	-0.825 (2.053)	0.688	4.484 (4.451)	0.314	-0.028 (0.325)	0.932	0.401 (1.043)	0.700	1.363 (0.810)	0.093	-0.244 (1.176)	0.836
**Race**													
White	2848	**REF**		**REF**		**REF**		**REF**		**REF**		**REF**	
Other	204	0.307 (0.652)	0.638	-0.178 (0.627)	0.776	0.115 (0.131)	0.380	-0.275 (0.200)	0.169	-0.329 (0.201)	0.102	0.073 (0.318)	0.819

**Body Mass Index** (per 1-unit increase)	3052	0.104 (0.028)	<0.001	0.059 (0.026)	0.023	0.008 (0.006)	0.152	-0.003 (0.009)	0.765	-0.024 (0.009)	0.007	0.006 (0.014)	0.690
**Smoker**													
Yes	80	**REF**		**REF**		**REF**		**REF**		**REF**		**REF**	
No	2972	-2.616 (1.021)	0.010	-2.908 (1.002)	0.004	-0.104 (0.187)	0.579	-0.353 (0.326)	0.279	0.258 (0.306)	0.399	0.959 (0.453)	0.034
**Marital Status**													
Married	2070	**REF**		**REF**		**REF**		**REF**		**REF**		**REF**	
Widowed	93	-0.655 (0.513)	0.202	-1.211 (0.497)	0.015	0.952 (0.194)	<0.001	-0.321 (0.257)	0.212	-0.505 (0.300)	0.093	-0.979 (0.394)	0.013
Separated/divorced	178	0.898 (0.551)	0.103	0.090 (0.493)	0.855	1.054 (0.153)	<0.001	-0.128 (0.216)	0.553	-0.577 (0.215)	0.007	-0.251 (0.289)	0.384
Never married	711	0.419 (0.481)	0.384	-0.184 (0.478)	0.700	0.966 (0.101)	<0.001	0.104 (0.159)	0.515	-0.641 (0.148)	<0.001	-0.621 (0.235)	0.008
**Employment Status**													
Employed	1767	**REF**		**REF**		**REF**		**REF**		**REF**		**REF**	
Retired	785	-0.326 (0.386)	0.399	-0.261 (0.354)	0.461	-0.032 (0.107)	0.765	-0.212 (0.182)	0.242	-0.393 (0.179)	0.028	-0.091 (0.270)	0.737
Other	403	1.729 (0.621)	0.005	1.800 (0.625)	0.004	0.060 (0.116)	0.606	0.78 (0.183)	<0.001	-0.005 (0.172)	0.979	-0.698 (0.287)	0.015
Unemployed	97	2.409 (1.005)	0.017	0.705 (1.030)	0.494	0.326 (0.191)	0.089	1.551 (0.289)	<0.001	-0.973 (0.283)	0.001	-1.477 (0.453)	0.001
**Chronic Illnesses**													
0	2163	**REF**		**REF**		**REF**		**REF**		**REF**		**REF**	
1	263	1.669 (0.537)	0.002	1.811 (0.529)	0.001	0.161 (0.110)	0.141	0.669 (0.185)	<0.001	-0.239 (0.171)	0.161	-0.900 (0.270)	0.001
2+	626	2.859 (0.362)	<0.001	3.177 (0.339)	<0.001	0.378 (0.081)	<0.001	0.719 (0.131)	<0.001	-0.204 (0.121)	0.091	-1.160 (0.198)	<0.001

b = adjusted unstandardized beta; SE = standard error.

Table S3. Tests of interactions between public health restrictions and physical activity change, sitting time change, and screen time change derived from linear regressions

	**Depression**		**Anxiety**		**Loneliness**		**Stress**		**Social Network**		**Positive Mental Health**	
**Interaction Term**	$F_{(6, 3022)}$	***p*-value**	$F_{(6, 3022)}$	***p*-value**	$F_{(6, 3022)}$	***p*-value**	$F_{(6, 3022)}$	***p*-value**	$F_{(6, 3022)}$	***p*-value**	$F_{(6, 3022)}$	***p*-value**
Public health restrictions X												
Physical activity change	0.54	0.775	0.21	0.973	0.64	0.702	0.35	0.911	0.95	0.459	0.25	0.960
Sitting time change	1.00	0.422	0.39	0.888	2.01	0.061	1.51	0.170	1.49	0.179	1.02	0.407
Screen time change	1.39	0.215	1.47	0.183	2.17	0.043	3.30	0.003	0.35	0.911	2.64	0.015

Statistical significance set at $p < 0.00278$
